# The first study on confirmation and risk factors of acute and chronic canine distemper in stray dogs in Wasit Province, Iraq, using enzyme-linked immunosorbent assay and reverse transcription-polymerase chain reaction

**DOI:** 10.14202/vetworld.2022.968-974

**Published:** 2022-04-18

**Authors:** Hadeel Asim Mohammad, Eva Aisser Ajaj, Hasanain A. J. Gharban

**Affiliations:** 1 Department of Internal and Preventive Medicine, College of Veterinary Medicine, University of Mosul, Nineveh, Iraq; 2 Department of Internal and Preventive Veterinary Medicine, College of Veterinary Medicine, University of Wasit, Wasit, Iraq

**Keywords:** epidemiology, Iraq, molecular, *Morbillivirus*, serology

## Abstract

**Background and Aim:**

In Iraq, stray dogs represent a critical population of free-roaming animals, which probably play a role in the transmission of different infections to other animals. Canine distemper is one of the most growing viral threats to carnivores in many countries worldwide, including Iraq. Therefore, this study was aimed to diagnose the disease using serological and molecular assay and the role of risk factors in the spreading infection.

**Materials and Methods:**

In all, 158 venous blood samples were collected randomly from stray dogs in rural and sub-urban areas of Iraq from May 2019 to December 2020. The samples were examined serologically using two enzyme-linked immunosorbent assay (ELISA) kits, immunoglobulin G (IgG) and immunoglobulin M (IgM), and molecularly by reverse transcription-polymerase chain reaction (RT-PCR) to detect and confirm chronic and acute infections. To determine the association between infection and various risk factors, the study animals were divided according to their locations, sexes, and ages. The age groups were ≤8 months (puppy), 1-3 years (young), and ≥3 years (old/mature).

**Results:**

ELISA result shows that 6.96% and 19.62% of dogs were seropositive for acute and chronic distemper, respectively. The titer of chronic infections (0.421±0.027) was significantly higher (p≤0.025) than that of acute canine distemper (0.337±0.016). On RT-PCR, 8.86% of dogs were found positive for distemper. Using RT-PCR as the gold standard, the sensitivity and specificity of the IgM ELISA kit were 75% and 98.63%, respectively, whereas the positive and negative predictivity were 81.82% and 97.96%, respectively. A significant variation (p<0.05) was observed in the distribution of positive findings among the different epidemiological risk factors. Compared with rural areas, positivity was significantly higher (p<0.05) in sub-urban areas on IgM (26.92%) and IgG (64.15%) ELISA and RT-PCR (34.62%). On IgM ELISA and RT-PCR, no significant differences (p>0.05) were found among the three age groups; however, positivity was significantly higher (p≤0.048) in the ≥3 years group (22.73%) on IgG ELISA. Furthermore, only IgG ELISA showed a significantly higher (p≤0.032) positivity rate in female dogs (25.23%) than in male dogs (7.84%).

**Conclusion:**

To the best of our knowledge, this is the first Iraqi study to demonstrate acute and chronic canine distemper in stray dogs, suggesting that the prolonged shedding of virus from positive dogs is a critical point in the epidemiology of the disease. Additional studies in dogs or other carnivores are required to establish baseline data on the prevalence of the disease in Iraq.

## Introduction

Canine distemper is a highly infectious and contagious disease in domesticated and wild dogs and several mammalian species in Canidae, Mustelidae, Procyonidae, Ursidae Viverridae, and Felidae families [1,2]. Morbillivirus of Family *Paramyxoviridae* is a causative agent of canine distemper, which is related antigenically to measles (in humans), rinderpest (in cattle and buffalo), and peste des petits ruminants (in sheep and goats) [3]. The disease has been recently recognized as a growing worldwide conservation threat to carnivores in many areas and countries [4]. Clinically, canine distemper is characterized by acute generalized symptoms, chronic localized and persistent infection of the central nervous system, or subclinical disease [5]. All breeds and ages of dogs can be affected, especially non-vaccinated ones through inhalation of infectious aerosols from recently infected (subclinical or diseased) animals or through contaminated food, water bowls, clothing, brushes, and other utensils [6,7].

Specific laboratory tests are usually unavailable to diagnose and clarify the prevalence of canine distemper or confirm a suspicion of infection. Furthermore, routine procedures are not helpful [8]. This routine investigation includes a culture of conjunctival or nasal swab samples, while post-mortem diagnostic methods include gross detection of pneumonia, digital hyperkeratosis, and tooth enamel hypoplasia which might non-specific lesions for distemper infection. In addition, histopathology can be used to detect necrosis and/or inclusion bodies in epithelial cells of internal organs. The variability of signs in dogs with distemper makes the clinical diagnosis difficult. Myoclonus appears to be the only neurological sign suggestive of distemper infection [9]. Although the isolation of the virus is essential, many obstacles are encountered in the isolation of the virus from affected dogs using tissue culture or in demonstrating the characteristic cytopathic effect of fusion formation [10]. However, enzyme-linked immunosorbent assay (ELISA) can be used as a simple, rapid, sensitive, and computerized serological test to detect acute and chronic canine distemper [11,12]. With the advances in molecular detection techniques, many assays have been described for canine distemper diagnosis with a varying degrees of sensitivity and specificity. Quantitative detection by reverse transcription-polymerase chain reaction (RT-PCR) has been approved as a useful tool for the rapid detection of canine distemper and the quantitative estimation of viral RNA in biological samples [13,14].

In Iraq, all stray dogs are unvaccinated, and infection control schemes are lacking. The control schemes include all control programs that are applied mainly to control the number of stray dogs as well as to prevent and stop the transmission of infections between dog populations or from dogs to other field animals, even humans, through biting or environmental contamination. Yet, the prevalence of the virus among the population of stray dogs has not been reported. Hence, the study aimed to confirm the prevalence of acute and chronic canine distemper in stray dogs using specific immunoglobulin M (IgM) and immunoglobulin G (IgG) ELISA kits and to confirm acute infection by RT-PCR. In addition, this study aimed to detect the association of PCR positivity with epidemiological risk factors (residence, age, and sex) in the study dogs.

## Materials and Methods

### Ethical approval

The present study was approved (approval no. 860-16/2/2019). by the Scientific and Ethical Committees of the Department of Internal and Preventive Veterinary Medicine, College of Veterinary Medicine in both University of Wasit (Wasit, Iraq) and University of Mosul (Nineveh, Iraq).

### Study period and location

The study was conducted from May 2019 to December 2020. The study was conducted on 158 stray dogs of different ages and sex from rural and sub-urban areas in Wasit Province, Iraq. The particular rural areas were Al-Battar, Al-Husayniah, Al-Sowadeh, and Al-Dujaily districts, whereas the sub-urban area was Al-Kut district. The samples were processed at the private Scientific Research Laboratory, AL-Qadisiya, Iraq.

### Samples and data

Approximately 5 mL of blood was collected from each dog from the cephalic vein using a disposable syringe. The sample was transferred equally between glass tubes with and without ethylenediaminetetraacetic acid (EDTA) gel as an anticoagulant and transported in cooled (4°C) condition to the laboratory. The anticoagulant-free tubes were centrifuged at 2200 × g for 5 min. The serum of each processed sample was divided into two labeled 1.5-mL Eppendorf tubes and frozen until used for serology. The EDTA tubes were immediately frozen for molecular assay.

In addition, data on the sex and age of the study dogs were documented as described by Tobias *et al*. [15]. Furthermore, the general health status of the study dogs was not confirmed, as this required unlimited time and budget, as well as the examiner protective measurement, which were not considered. However, the general clinical observation was that the animals were relatively healthy. To determine the association between infection and various risk factors, the study animals were divided according to location, sex, and age. The age groups were ≤8 months (puppy), 1-3 years (young), and ≥3 years (old/mature).

### Serology by ELISA

Two types of qualitative monoclonal ELISA kits (Demeditec Diagnostics, Germany) were used in this study. One targeted IgM antibodies (DE2479) to diagnose acutely infected dogs, and the other was used to detect IgG (DE2478) antibodies to identify chronically infected dogs. The reagents, buffers, positive and negative controls, and sample sera were prepared and diluted following the manufacturer’s steps. The assays were performed, and the results were interpreted at an optical density (OD) of 450 nm using an ELISA reader (BioTek, USA). The OD values of the positive and negative controls and the samples were validated and interpreted to evaluate for positivity. In addition, the ODs of positive samples detected by IgG and IgM ELISA were considered as the titers for infection severity.

### Molecular assay by RT-PCR

#### Extraction

Total RNAs were extracted from the EDTA-treated blood samples according to the manufacturer’s instructions for *AccuZol™* reagent kit (Bioneer, Korea). Briefly, 250 μL blood sample was added to 750 μL AccuZol™ in a 1.5-mL Eppendorf tube and suspended several times by vortexing. Chloroform (200 μL) was added to each sample, and the mixtures were vortexed vigorously for 15 s, incubated on ice for 5 min, and centrifuged at 16,000 × g for 15 min at 4°C. The resulted supernatant was aspirated and placed into 1.5 mL sterile tube, and an equal volume of isopropyl alcohol was added. The mixture was inverted, incubated at −20°C for 10 min, centrifuged at 16,000 × g for 10 min, added with 1 mL of 80% ethanol, and mixed again by vortexing. After centrifuging again at 16,000 × g for 5 min at 4°C, the supernatant was removed, and the pellet of RNAs retrieved was dissolved in RNase-free water, incubated at 60°C for 10 min, and deep-frozen.

### cDNA synthesis

The RNA was reverse-transcribed into cDNA using an AccuPower RocketScript™ Cycle RT PreMix kit (Bioneer) at 20-mL final volume. After cDNA amplification, the products were stored at −20°C for further molecular analysis.

### PCR amplification

An ExicyclerTM 96 Real-Time Quantitative Thermal Block (Bioneer) system was used to amplify cDNA using both the designed (CDVF: 5’-CAC CTT CTA CAA CGA GCT GCG-3’ and CDVR: 5’-ATC TTC TCA CGG TTG GCC TTG-3’) and provided primer (Macrogen, Korea) of the NP gene and housekeeping gene b-actin [16].

In Real-Time PCR, the Onderstepoort strain of canine distemper virus cultured on Vero cells was used as a positive control. This strain was obtained from the Private Scientific Research Laboratory (AL-Qadisiya, Iraq). The extracted total RNA from Vero cells infected with the Onderstepoort strain of canine distemper virus and ultra-pure water was used as positive and negative control samples, respectively. The PCR conditions were performed as follows: One cycle for initial denaturization (94°C, 5 min), 40 cycles for denaturization (94°C, 20 s), and annealing/extension (60°C, 45 s). The amplification product had a length of 93 bp. The threshold cycle (Ct) was calculated as described previously [17].

### Statistical analysis

Data were analyzed using the GraphPad Prism version 6.01 software (GraphPad Software Inc. USA). The Chi-square (χ^2^) test and odds ratio were used to express significant differences between positive findings of the diagnostic assays and to determine the association between positive results and epidemiological risk factors at a significance level of p<0.05 [18].

## Results

### Serology

**Figure-1:**
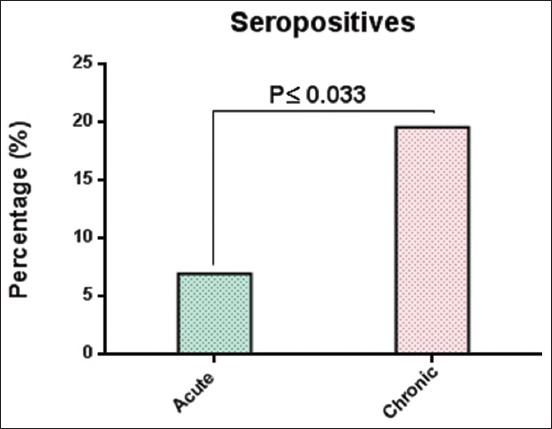
Total results of acute and chronic canine distemper among 158 stray dogs.

**Figure-2:**
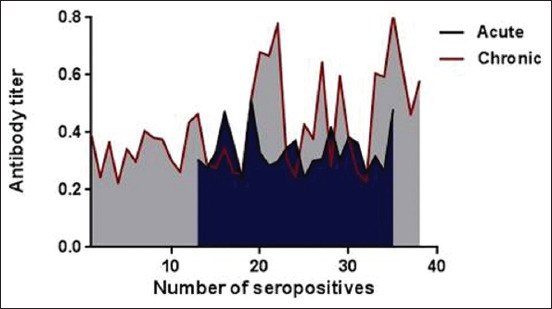
Titers of immunoglobulin M and immunoglobulin G ELISA kits used for detection acute and chronic infections, respectively.

ELISA showed that 6.96% (11/158) and 19.62% (31/158) were significantly (p≤0.013) (Figure-1 and Table-1) seropositive for acute and chronic canine distemper infections. The titers of antibodies differed significantly (p<0.05) between both infections. However, the titer (mean±standard error [SE]) for chronic infections (0.421±0.026) was significantly higher (p≤0.025) than that for acute infections (0.337±0.024) (Figure-2).

**Table 1:** Total results of testing 158 dogs by ELISA Kits and RT-PCR.

Result	Total positive	

	No.	%
IgG (Chronic sero-infection)	31	19.62
IgM (Acute sero-infection)	11	6.96
RT-PCR (Acute molecular infection)	14	8.86

IgM=Immunoglobulin M, RT-PCR=Reverse transcription polymerase chain reaction, IgG=Immunoglobulin G

**Figure-3:**
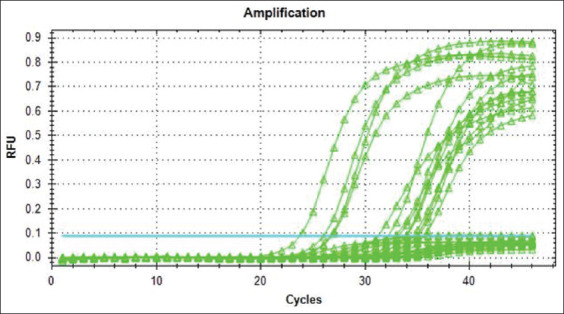
Amplification reaction of positive samples at a Ct value of 21.75-27.75.

On RT-PCR, 8.86% (14/158) of dogs tested positive (Figure-3). Using RT-PCR as the gold standard of testing, the sensitivity and specificity of IgM ELISA were 75% and 98.63%, respectively, whereas the positive and negative predictivity were 81.82% and 97.96%, respectively.

Significant differences (p<0.05) were found in the positive findings among the different epidemiological risk factors (Table-2). Compared with rural areas, positivity was significantly higher (p<0.05) in sub-urban areas on IgM (26.92%) and IgG (64.15%) ELISA and RT-PCR (30.77%). On IgM ELISA and RT-PCR, no significant differences (p>0.05) were found among the three age groups; however, positive findings were significantly higher (p≤0.048) in the ≥3 years group (22.73%) on IgG ELISA. Furthermore, only IgG ELISA found a significantly higher (p≤0.032) positivity rate in female dogs (25.23%) compared with male dogs (7.84%).

**Table 2:** Distribution of positive results related to epidemiological risk factors.

Factor	Total No.	Test		

		ELISA		RT-PCR

		IgM	IgG	
Region				
Rural	132	4 (3.03%)	19 (14.39%)	5 (3.79%)
Sub-urban	26	7 (26.92%)*	12 (64.15%)*	9 (34.62%)*
p-value		0.043	0.036	0.044
Age				
∼≤8 Months	23	2 (8.7%)	5 (21.74%)	2 (8.7%)
∼1–3 Years	91	5 (5.49%)	16 (17.58%)	7 (7.69%)
∼≥3 Years	44	4 (9.09%)	10 (22.73%)*	5 (11.36%)
p-value		0.052	0.048	0.062
Sex				
Females	107	8 (7.48%)	27 (25.23%)*	11 (10.28%)
Males	51	3 (5.88%)	4 (7.84%)	3 (5.88%)
p-value		0.08	0.032	0.051

Significance

* (p<0.05), IgM=Immunoglobulin M, RT-PCR=Reverse transcription polymerase chain reaction, IgG=Immunoglobulin G

## Discussion

Canine distemper is a systemic disease in carnivores, which may result in high mortalities among stray dogs. Additional data on disease prevalence and a better understanding of disease ecology in wild populations need to be acquired. Specific serological assays for measuring IgM and/or IgG in serum samples are important to determine an acute or chronic (carrier) stage of infection [11,19,20]. The two types of indirect ELISA in the present showed that 6.96% and 19.62% of dogs were seropositive for antibodies IgM and IgG against canine distemper virus, respectively. Elevated antibody-titers can be detected serologically for several months after subclinical or clinical infection, and the virus-specific IgM could persist for at least 3 months after infection [3,21]. However, the class of IgM antibody produced early in viral infection indicates ongoing or recent viral multiplication [22]. In contrast, IgG seropositivity indicates previous exposure to the virus, which is possible since stray dogs may not have been vaccinated [23]. The seroprevalence of canine distemper antibodies is 7.5% in Nigeria [24], 9-72% in India [25,26], 9.03% in Turkey [27], 15% in Brazil [28], 17.52% in Iran [29], and 18.7% in Spain [30]. However, information on the diagnosis and prevalence of distemper infection in dogs is relatively scarce, and most reports have been based on the clinical manifestation in the suspected dogs. The limited data could be due to the difficulty of culturing the virus, the time required for the virus to grow in the cell lines, or the possibility of the virus to spread in the environment and promote infection [31]. The outcome and severity of clinical signs could vary markedly with strain virulence, age of the animal, and the immune status of the animal, which is crucial to the clearing or persistence of the virus [3]. ELISAs have been developed based on recombinant proteins to detect canine distemper virus infections using specific markers [21]. The high sensitivity and specificity of ELISA were indicated by the detection of IgM antibodies and comparison of tested sera results with IgG antibodies. Immunological resistance and sensitivity of a dog to canine distemper virus are multifactorial, and the predictive value of antibody-responses or antibody-titers can be challenging due to variations in strain virulence, infective viral dose, adequacy of helper T cell-mediated immunity, immune-mediated cytotoxicity, and the persistence of memory cells [32,33].

In this study, we provide the first genetic evidence of canine distemper in Iraq, which was achieved by RT-PCR in confirming acute infection in blood samples. The positivity rate of this technique in our study was 8.86%. In other studies, the molecular positivity was 15% in Iran [34], 21% in India [35], 24.88% in China [36], 30.66% in Hungary [37], and 73.74% in Argentina [38]. Many studies have confirmed the high sensitivity and specificity of RT-PCR in the detection of canine distemper virus in different clinical samples, including serum, urine, and conjunctival swabs, as well as in confirming sub-acute and chronic stages of the disease in cases of poor viral shedding [35,39-40]. Determining the prevalence of infection among stray dogs using different diagnostic techniques was deemed necessary in the present study, given the absence of infection prevention and control schemes. A significant positivity rate on IgG ELISA suggests either previous exposure of the animals to the virus or a high prevalence of chronic infection among the stray population.

The significant variation in the incidence of canine distemper in dogs between rural and sub-urban areas was in agreement with a study by Frölich *et al*. [41] that found higher canine distemper prevalence in sub-urban areas but were incompatible with study by Ashmi *et al*. [35] that showed a higher infection incidence in rural areas. However, a higher positivity in dogs in sub-urban areas suggests either abundant viral contamination of the sub-urban environment or an emerging role of urban domestic dogs as maintenance hosts for canine distemper. Nevertheless, carnivores in rural areas might act as direct viral sources for dogs in both sub-urban and rural areas [28].

In our study, a significant association (p<0.05) was found between positivity and the age of the study. Our results suggest that stray dogs of different ages are exposed to similar rates of infection. Conversely, a higher seroprevalence of IgG antibodies in adult dogs could be due to increased disease exposure with age, constant force of infection in endemic areas, and differential rates of exposure in a population experiencing sporadic outbreaks [42]. However, the age-related seroprevalence of canine distemper might be debatable [43]. A study by Bergmann *et al*. [44] reported that the survival of canine distemper virus in a stray dog population was constant at different ages, whereas Temilade *et al*. [24] demonstrated that the disease onset was more likely within the period from birth until 2 years of age. Furthermore, de Almeida Curi *et al*. [28] suggested that positivity is unrelated to age and that titers of antibodies were greater in adult dogs than in puppies. In fact, the majority of puppies acquire maternal immunity through placenta or colostrum, and the low prevalence of IgG antibodies in this age group could be due to a lack of maternal immunity or poor immune competency for the acquired immunity at this age [25].

Concerning the sex factor, no significant differences were observed between the positive females and males by the serological and molecular assays. In comparison between our diagnostic assays, positive results of females were significantly higher by IgM ELISA and RT-PCR than IgG ELISA. (p<0.05). In other studies, Kim *et al*. [45] found no significant role of sex in the susceptibility of animals to canine distemper. However, Buragohain *et al*. [46] reported that male dogs have a higher susceptibility to canine distemper. In contrast, Temilade *et al*. [24] found a higher disease prevalence in female than in male dogs. We hypothesized that female dogs may experience higher rates of stress due to reproductive and hormonal reasons. Furthermore, the method of selecting the study dogs may have negatively impacted the findings of the present study.

## Conclusion

In Iraq, acute and chronic infections of canine distemper are prevalent in stray dogs, suggesting that the prolonged shedding of the virus from positive dogs is a critical point in the epidemiology of the disease. Both ELISA and RT-PCR showed high sensitivity and specificity in detecting infection. Stray dogs in sub-urban areas may act as a reservoir of pathogens for rural carnivores, including dogs. Furthermore, our findings suggest that the virus may circulate in both sub-urban and rural stray dogs. Further studies in dogs or other carnivores are required to establish baseline data on the prevalence of the disease in Iraq.

## Authors’ Contributions

HAJG: Sample collection and serological examination of sera. EAA and HAM: Molecular detection and statistical analysis. All authors read and approved the final manuscript.
